# Male genital lichen sclerosus in a pediatric case: A focus on the reflectance confocal microscopy presentation

**DOI:** 10.1111/srt.13304

**Published:** 2023-03-16

**Authors:** Lixin Chen, Ying Wang, Ji Wang, Bei Qin, Qinfeng Li

**Affiliations:** ^1^ Department of Dermatology Tianjin Children's Hospital Tianjin China

1

An 8‐year‐old boy presented to our department with asymptomatic, whitish plaques on the glans for 6 months. He had experienced no other discomfort, urinary stream narrowing, or dysuria; however, his rash worsened. The patient was generally healthy and had no known underlying disease or relevant family history of similar skin lesions. The physical examination revealed a redundant prepuce and clearly demarcated porcelain‐white atrophic plaques on the glans, coronal sulcus, and foreskin, without erosions (Figure 1a, b). Laboratory investigations revealed normal values of the complete blood count, urinalysis results, liver and renal functions, antinuclear antibody, immunoglobulin (Ig), anti‐double‐stranded DNA, thyroid function test, and hepatitis test.

**FIGURE 1:**
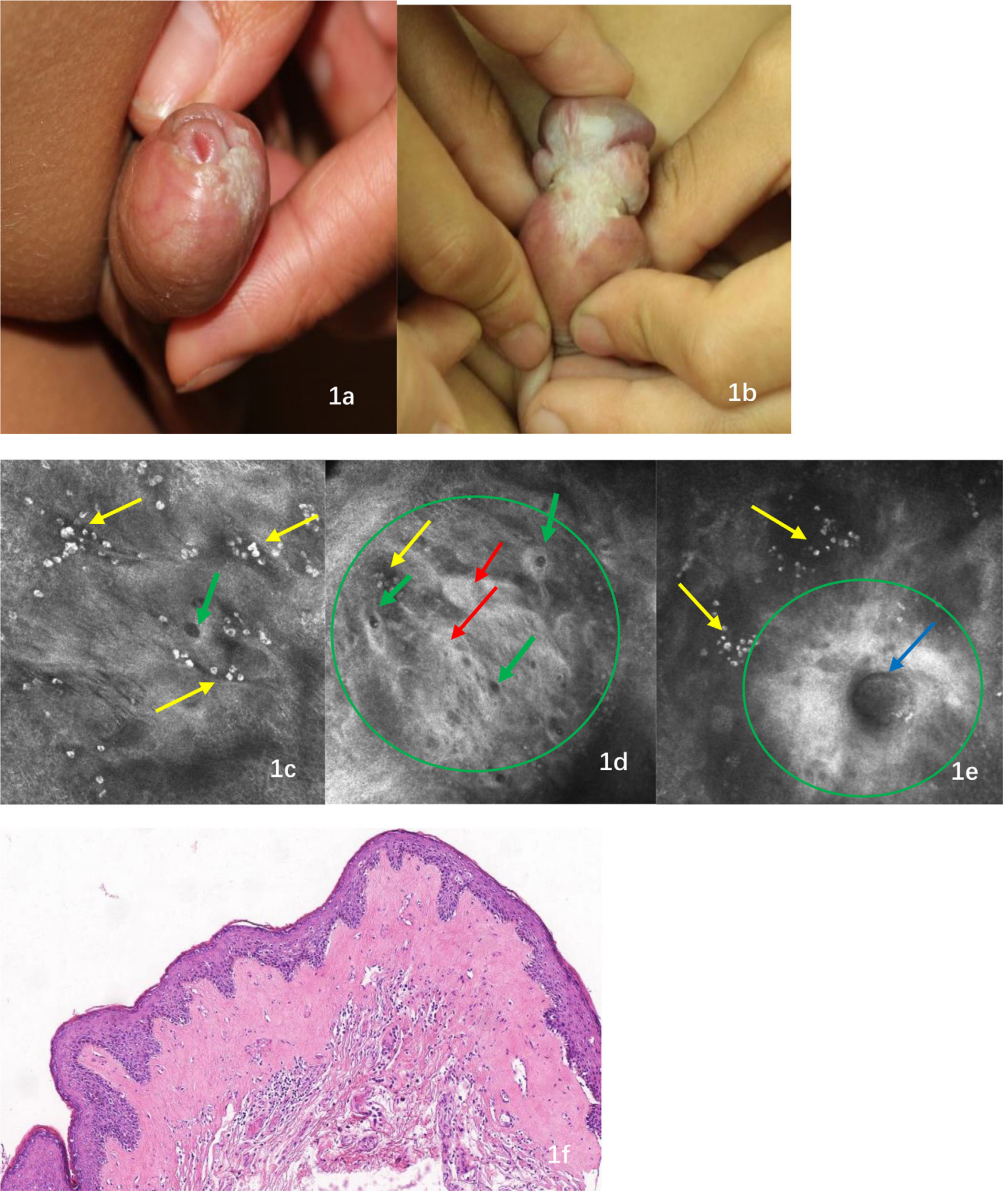
The clinical, RCM characteristics, and histopathological of male genital lichen sclerosus (MGLSc) in the pediatric. Clinical presentation (1a and 1b) shows clearly demarcated porcelain‐white atrophic plaques. RCM examination (1c–1e) shows white, coarse fibrous material with a diffusely present distribution (green oval), and some were gathered in a fascicular distribution (red arrow); numerous highly refractive round cellular structures (yellow arrow) and low‐refractive dilated round structures with a scattered distribution (green arrow); round cyst‐like structures containing medium‐low‐refractive substances (blue arrow). Histopathology images (1f) show epidermal atrophy, liquefaction degeneration of basal cells, and the homogenization of collagen in the superficial dermis.

Based on the patient's clinical manifestation and progression, we suspected lichen sclerosus and performed noninvasive reflectance confocal microscopy (RCM). The findings were as follows (Figure 1c‐e): Epidermal examination revealed a disorganized epidermal structure, significantly reducing the number of epidermal scanned layers, and round cyst‐like structures containing medium‐low‐refractive substances. At the dermo‐epidermal junction, the dermal papillary rings were destroyed and appeared nonrimmed. As the image depth increased, the superficial dermis was filled with medium refractivity, as well as coarse fibrous material, and some of the material was gathered in fascicular patterns. Numerous high‐refractive uniformly round cells and larger irregular cellular structures were diffusely distributed and occasionally aggregated. A large number of dilated low‐refractive grand or canalicular structures were vertically arranged on the confocal sections. Histopathological examination (Figure 1f) showed epidermal atrophy, liquefaction degeneration of basal cells, bands of homogenization of collagen and lymphocytes, and dense infiltration of melanophages.

Based on the clinical and histological findings, the diagnosis of male genital lichen sclerosus (MGLSc) was made.

MGLSc is a rare acquired, chronic inflammatory dermatosis in which persistent exposure of the susceptible epithelium to urinary occlusion is the most common pathological mechanism.^1^, ^2^ Clinically, the predominant symptoms of MGLSc are porcelain‐white atrophic plaques on the glans, coronal sulcus, and foreskin. Most mature men have symptoms associated with spontaneous itching, burning, bleeding, and male dyspareunia; however, in pediatric patients, MGLSc is often asymptomatic and easily leads to an ignored and missed diagnosis. Since the disease has a progressive course and causes architectural changes in the genitalia, dysuria, scarring, architectural changes, and squamous cell carcinoma of the penis,^1^, ^2^ a seasonable and accurate diagnosis is urgently required.

RCM can noninvasively observe changes in skin cell levels, and previous studies have suggested a high corresponding relationship between RCM images and histopathological features in the LS of females and male adults.^3^, ^4^, ^5^ However, few studies have investigated pediatric MGLSc. In our study, the RCM images of the boy revealed major key diagnostic features of LS, including a significantly reduced number of scanned layers, the destruction of dermal papillary rings, numerous cellular structures, coarse fibrous material, and the diffuse distribution of dilated canalicular structures in the upper dermis. This information and these clues confirmed that although the physiological structures of boys and girls differ greatly, the key points for diagnosing LS by RCM are unanimous, and this report further strengthens the evidence that RCM can be used to visualize major key diagnostic features of MGLSc. We recommend RCM as a useful adjuvant tool for diagnosis in children, but larger case series remain to be further studied.

## CONFLICT OF INTEREST STATEMENT

The authors declare no conflicts of interest.

## ETHICS STATEMENT

Approval for this study was obtained from the Ethics Committee of Tianjin Children's Hospital (Ref. No. KJRCPYXM2020‐02). The parents signed an informed consent form to participate in the study.

## Data Availability

The data that support the findings of this study are available on request from the corresponding author. The data are not publicly available due to privacy or ethical restrictions.
